# Pre-Operative Predictors of Concurrent Choledocholithiasis in Patients With Acute Calculus Cholecystitis: An External Validity Analysis

**DOI:** 10.7759/cureus.11039

**Published:** 2020-10-19

**Authors:** Saif Affas, Abdelkader Chaar, Jose Zamora-Sifuentes, Nouf Turki, Abdulla Nasser, Susan Szpunar, Mohammed Barawi

**Affiliations:** 1 Internal Medicine, Ascension St. John Hospital, Detroit, USA; 2 Internal Medicine, Yale-New Haven Hospital, New Haven, USA; 3 Gastroenterology and Hepatology, George Washington University, Washington, USA; 4 Biomedical Investigations and Research, Ascension St. John Hospital, Detroit, USA; 5 Gastroenterology, Ascension St. John Hospital, Detroit, USA

**Keywords:** pre operative evaluation, acute calculus cholecystitis, dilated common bile duct, choledocholithiasis

## Abstract

Introduction

Patients that are presented with acute calculus cholecystitis (AC) and elevated liver enzymes markers (LEM), often require evaluation for concurrent choledocholithiasis (CDL). Currently, evaluation guidelines follow the American Society of Gastroenterology Endoscopy (ASGE) recommendations.

Objectives

The aim of the study was to externally validate both ASGE and the Chisholm predictors in a community hospital patient cohort.

Methods

We conducted a retrospective study of patients who presented to Ascension Saint John hospital with AC and elevated LEM over a period of two years. Sensitivity (SEN), specificity (SP), positive predictive value (PPV) and negative predictive value (NPV) were used to test the external validity of ASGE and Chisholm algorithms.

Results

A total of 132 patients’ charts were reviewed, and 87 patients included. Chisholm predictors SEN, SP, PPV and NPV were 50%, 82%, 18%, and 95% respectively versus 100%, 19%, 8%, 100% for the ASGE predictors model. In the ASGE module, SP and PPV can be significantly improved to 60% and 13%, respectively, by changing a few risk categories including age and LEM range.

Conclusions

External validation of the Chisholm module in our patient cohort showed that it would lead to a low referral rate for unnecessary imaging and thus might be more cost-effective, especially when compared to current ASGE recommendations which would have a higher referral rate. On the other hand, current ASGE recommendations successively labeled all the patients with CDL, while the Chisholm module missed around 50 percent. We also observed that with the current ASGE module, the referral rate for further imaging and diagnostic tests can be possibly improved by adjusting a few of the predictors including the age and the abnormal liver transaminases range, but this observation is arbitrary and will need to be validated in a larger cohort study.

## Introduction

Patients presenting with acute calculus cholecystitis (AC) and markedly elevated liver enzymes often require evaluation for concurrent choledocholithiasis (CDL). Preoperative evaluation is usually done with endoscopic ultrasound (EUS), magnetic resonance cholangiopancreatography (MRCP), endoscopic retrograde cholangiopancreatography (ERCP), or by intraoperative cholangiogram (IOC) [1-3]. These tests are expensive, some are invasive, and they often delay definitive care [4-6].

Guidelines from the American Society for Gastrointestinal Endoscopy (ASGE) divided patient into four risk groups:

Very strong risk group, which includes any of the following: reporting common bile duct (CBD) stone on trans-abdominal ultrasound (US), clinical ascending cholangitis, or bilirubin >4 mg/dl.

Strong risk group, which includes any patient that has dilated CBD on US (>6 mm) and bilirubin level between 1.8-4 mg/dl, together.

Moderate risk group which includes any patient with an abnormal liver biochemical test other than bilirubin, age older than 55 years, or clinical gallstone pancreatitis.

Low-risk group which includes any patient with none of the above predictors.

The ASGE guideline suggests that: any patient in the very strong and strong risk group should be referred for ERCP. On the other hand, patients in the moderate risk group for CDL should undergo further diagnostic studies to exclude concomitant CDL, while any patient in the low-risk group can be referred directly for surgical cholecystectomy [7].

These guidelines have been limited by poor performance, evidenced by retrospective validation studies that showed unnecessarily high referral rates for further diagnostic studies [8-9].

Chisholm et al. recently published a novel module that included new predictors along with a new management algorithm [10]. The new module included three predictors, a common bile duct diameter larger than 6 mm, alkaline phosphatase (ALP) above the normal range, and alanine aminotransferase (ALT) greater than three times the normal level. According to these predictors, patients with AC can be categorized into three risk groups for having concomitant CDL: high-risk group (positive all the three predictors), intermediate-risk group (positive two out of the three predictors), and low-risk group (one or none of the risk predictors). The new management algorithm suggested that patients in the high-risk group can be referred directly to ERCP without further imaging or delay, while patients in the moderate-risk category should undergo further imaging to decide the management plan. On the other hand, patients in the low-risk group can proceed to cholecystectomy with no need for further imaging or ERCP [10].

They validated the new model on a cohort of 108 patients and compared results with the current ASGE recommendations.

When compared with the current ASGE guidelines, the new model classified about 70% of the cohort as low risk with the suggestion to proceed to cholecystectomy without further testing; of these only 1.4% would have CBD stones. Patients with all three risk factors (high risk for CDL, approximately 10% of the validation cohort) had negative ERCP 22% of the time. On the other hand, applying current ASGE guidelines to the same cohort showed a negative ERCP proportion of 42.4%, and it also showed that 62.6% of the patients would be referred for further testing despite not having CDL [10].

To date, the applicability of the above-mentioned findings has not been tested in a community hospital setting, where patient characteristics and practice patterns can be very different from the academic setting in which the Chisholm model was developed and validated. Given the discrepancy between the ASGE guidelines and the new recommendation from the Chisholm et al. study, the purpose of this study was to test the external validity of both models in our patient cohort in a community hospital setting.

## Materials and methods

We conducted a retrospective chart review of all adult patients who presented to Ascension Saint John Hospital with AC during the period 1/1/17-12/31/18. We identified patients ICD-9 and ICD-10 codes associated with acute cholecystitis. We included all patients with proven acute calculus cholecystitis (decided by surgeon, pathology report, or hepatobiliary iminodiacetic acid [HIDA] scan). Patients who did not have documented imaging, common bile duct diameter, or liver enzymes were excluded. Furthermore, any patients with prior procedures that involved the common bile duct (CBD stenting/sphincterotomy of the ampulla of vater) were also excluded.

For all patients, the following variables were collected: age at time of diagnosis, sex, race (White vs Black/African American (AA)), aspartate aminotransferase (AST), alanine aminotransferase (ALT), alkaline phosphatase (ALP), total bilirubin, common bile duct (CBD) diameter, possible CBD stone on initial imaging, suspected gallstone pancreatitis and suspected ascending cholangitis. Choledocholithiasis was defined as any reported biliary stone or CBD sludge on imaging or found post-operatively. The data were analyzed using Statistical Package for the Social Sciences (SPSS) version 26.0 (IBM Corp., Armonk, NY). Mean and standard deviation (SD) were reported for normal distributed continuous variables, median and range were reported for non-normal distributed continuous variables, while frequencies were reported for categorical variables. Sensitivity, specificity, positive predictive value, and negative predictive value were calculated and compared for both models.

## Results

We initially reviewed 132 charts; 87 patients met the inclusion criteria. The most common reason for exclusion was the absence of acute calculus cholecystitis. Other exclusion criteria included the lack of documented CBD diameter, previous CBD manipulation, or patients who did not have the surgery and left with no documented evaluation or follow-up regarding the presence of choledocholithiasis.

Regarding the ASGE model, patients in the very strong, strong, or moderate risk categories were considered as a positive test, while everyone else was considered a negative test. For the Chisholm model, patients with three out of three predictors and two out of three predictors were considered a positive test, while everyone else was considered negative.

The majority of patients were females (70%), with a race distribution of around 1 to 1 ratio of Black/African American to white patients, the mean age was 56 ± 18 years. Median (range) levels of AST, ALT, and ALP were 33 (10-866) U/L, 33 (9-823) U/L, and 84 (17-711) U/L, respectively. The mean total bilirubin was 1 ± 1.09 mg/dl. Twenty-two patients had abnormally dilated CBDs (25%), three patients had a suspected CBD stone noticed on initial imaging, six patients had suspected gallstone pancreatitis and two patients had suspected ascending cholangitis (Table 1).

**Table 1:** Characteristics of patient cohort M: Male, F: Female, AA: African American, ALT: Alanine aminotransferase, AST: Aspartate transaminase, ALP: Alkaline phosphatase, CBD: Common bile duct.

Variable	Number (total number = 87)	Mean % or Median/Range
Age		56 ± 18 year
Sex	61 F	70% F
	26 M	30% M
Race	42 White	48% White
	45 AA	51% AA
AST		33 (10-866) U/L
ALT		33 (9-823) U/L
ALP		84 (17-711) U/L
Total Bilirubin		1.0 ± 1.09
Abnormal CBD diameter	22	25%
Suspected ascending cholangitis	2	2%
Suspected CBD stone on initial imaging	3	3%
Suspected gallstone pancreatitis	6	6%

Six patients had acute calculous cholecystitis with concomitant choledocholithiasis. Table 2 shows further characteristic for each of these patients.

**Table 2:** Patients with acute cholecystitis and concomitant choledocholithiasis CBD-D: Common bile duct diameter, CBD-S: Common bile duct stone on initial imaging, T. Bili: Total bilirubin, G.S. panc.: Suspected, Gallstone pancreatitis, A. chol: Suspected ascending cholangitis, AA: African American, AST: Aspartate transaminase, ALT: Alanine aminotransferase, ALP: Alkaline phosphatase.

Race	Sex	Age	AST	ALT	ALP	CBD-D	CBD-S	T. Bili	GS Panc.	A. chol.
White	M	86	597	375	222	7mm	no	3.6	yes	no
AA	F	66	39	37	711	6mm	no	2	no	no
AA	F	65	519	411	133	5mm	no	2.6	no	no
AA	F	19	157	85	95	8mm	possible	0.7	no	no
White	F	22	35	120	160	9mm	possible	1.1	no	no
White	F	69	525	246	80	6mm	no	4.1	no	no

Assessing the risk for CDL

By applying the ASGE predictors to our cohort, 11 (12.6%) patients were in the high-risk group for which they would be referred to ERCP. Sixty patients (68.9%) were labeled as moderate risk, meaning they would need further testing before undergoing definitive treatment. Sixteen (18.3%) patients fell in the low-risk category. The ASGE predictors successfully labeled all patients with CDL as either high or moderate risk groups. Accordingly, when the diagnosis of choledocholithiasis was considered an endpoint, the sensitivity (SEN), specificity (SP), positive predictive value (PPV), and negative predictive value (NPV) for this model were 100%, 19%, 8%, and 100%, respectively.

On the other hand, the newer model by Chisholm would refer only 15 (17.2%) patients for further imaging, while 70 (80.4%) patients would fall in the low-risk category. Only two (2.2%) patients were considered high-risk for CDL. This model failed to identify three patients who had concomitant CDL as high or moderate risk, which means these patients would undergo cholecystectomy without further imaging or ERCP to rule out CDL first (Table 3).

**Table 3:** Number of patients in each risk group when assessed by either model

Risk group category	ASGE predictors	Chisholm predictors
Low-risk group	16	70
Moderate-risk group	60	15
High-risk group	11	2

Accordingly, when diagnosis of choledocholithiasis was considered an endpoint, the SEN, SP, PPV, and NPV for this model were 50%, 82%, 18%, and 95%, respectively.

Reporting our observation

We observed that the ASGE predictors can be significantly enhanced by adjusting a few variables in the moderate-risk group. These include moving the age greater than 55 years from the moderate-risk group to the low-risk group and increasing the abnormal liver transaminase range to be at least greater than three times the normal limit to be included in the moderate risk group (Table 4).

**Table 4:** Possible adjusted ASGE predictors ASGE: American Society of Gastroenterology Endoscopy, CBD: Common bile duct, AST: Aspartate aminotransferase, ALT: Alanine aminotransferase.

Risk group	Predictors
Very strong risk	
	CBD stone on transabdominal ultrasound (US)
	Clinical ascending cholangitis
	Bilirubin >4 mg/dl
Strong risk	
	Dilated CBD on US (>6 mm)
	Bilirubin level 1.8-4 mg/dl
Moderate risk	
	AST/ALT> three times the upper limit or any abnormal Alkaline Phosphatase
	Clinical gallstone pancreatitis
Low risk	
	Any patient with none of the above predictors

This would result in an observed drop in the number of unnecessary referrals to imaging when applied to our cohort (Table 5).

**Table 5:** Number of patients in each risk group when assessed by current ASGE predictors and with the observed adjusted ASGE predictors ASGE: American Society of Gastroenterology Endoscopy

Risk group category	ASGE predictors	Adjusted ASGE predictors
Low-risk group	16	70
Moderate-risk group	60	33
High-risk group	11	43

After applying these modifications, the SEN, SPE, PPV, and NPV for the adjusted ASGE model were 100%, 60%, 13%, and 100%, respectively.

## Discussion

The diagnosis of CDL in a patient presenting with acute calculus cholecystitis along with elevated liver enzymes is a challenging dilemma. The current management algorithm and risk stratification have been mainly driven by the American Society of Gastroenterology recommendations. Recent studies have shown that these recommendations contribute to a delay in reaching definitive management, which increases costs because of referral for unnecessary imaging [10].

Chisholm et al. [10] have suggested an internally validate new risk stratification according to mainly three predictors which are: any elevated alkaline phosphatase, common bile duct diameter greater than 6 mm, and ALT elevation three times greater than the normal level.

In this study, we examined the external validity of the two different models to predict CDL in patients with AC using a cohort of 87 patients who presented to our community hospital. By external validation of the ASGE predictors, we found that it had 100% sensitivity, 19% specificity, 8% positive predictive value, and 100% negative predictive value. Given the low specificity, ASGE predictors led to a high referral rate for seemingly unnecessary diagnostic imaging and further workup before reaching definitive management. It, however, had a 100% sensitivity and did not miss any patient that had AC with CDL.

On the other hand, external validation of the newer Chisholm predictors showed a lower referral rate for imaging before cholecystectomy. These predictors had an SPE of 82% and an NPV of 95% when applied to our cohort. In contrary to the ASGE predictors, we observed an SEN of 50% and PPV of 18%. This model mislabeled half the patients that had AC with CDL as low risk.

We also observed, in our patient cohort, that the ASGE predictors can possibly be improved and the unnecessary imaging referral rate can potentially be lowered by adjusting the age category and the abnormal liver transaminases range. These modifications, however, were arbitrary and will require further validation in future studies on a larger and multicentral cohort. Of note, to our knowledge, our study represents the first external validation of the Chisholm model in a community hospital setting. Patients in our cohort might have significantly different baseline features than the patients in the Chisholm et al. cohort which originated from an academic setting. Moreover, our practice pattern and referral are significantly different from the Chisholm study. All these differences highlight the importance of testing the external validity of both the ASGE and Chisholm et al. models to understand their performance in the community hospital setting before applying them in clinical patient management.

## Conclusions

External validation of the current ASGE guidelines in our patient cohort showed a high referral rate for unnecessary imaging which could increase the cost of diagnosis before definitive management, that been said, it effectively labeled all patient with possible AC with CDL. On the other hand, external validation of Chisholm’s predictors in our patients' cohort showed a low referral rate for unnecessary imaging, but it mislabeled half of the patients who had CDL. We also observed, that the current ASGE predictors can be improved and the unnecessary imaging referral rate can potentially be lowered by adjusting few variables as removing the age category from a medium risk and increase the abnormal liver transaminases range to above three times the normal limit. These modifications, however, were arbitrary and will require further validation in larger and multicentral cohort to insure safely triaging and management of patients presenting with acute calculus cholecystitis.
